# *Pseudidiomarina fusca* sp. nov., Isolated from the Surface Seawater of the Western Pacific Ocean

**DOI:** 10.3390/microorganisms12020408

**Published:** 2024-02-18

**Authors:** Yaru Wang, Xiaolei Wang, Xueyu Gao, Jingjing He, Xiaoyu Yang, Yunxiao Zhang, Xiaohua Zhang, Xiaochong Shi

**Affiliations:** 1 Frontiers Science Center for Deep Ocean Multispheres and Earth System, College of Marine Life Sciences, Ocean University of China, Qingdao 266003, China; yaru0118@126.com (Y.W.); wangxiaolei@ouc.edu.cn (X.W.); gaoxueyu@stu.ouc.edu.cn (X.G.); hejingjing@stu.ouc.edu.cn (J.H.); xiaoyuyang0828@126.com (X.Y.); zyunxiao199999@163.com (Y.Z.); xhzhang@ouc.edu.cn (X.Z.); 2 Laboratory for Marine Ecology and Environmental Science, Laoshan Laboratory, Qingdao 266237, China; 3 Institute of Evolution & Marine Biodiversity, Ocean University of China, Qingdao 266003, China

**Keywords:** *Pseudidiomarina fusca* sp. nov., surface seawater, western Pacific Ocean, polyphasic taxonomy

## Abstract

The Gram-negative marine bacterium GXY010^T^, which has been isolated from the surface seawater of the western Pacific Ocean, is aerobic, non-motile and non-flagellated. Strain GXY010^T^ exhibits growth across a temperature range of 10–42 °C (optimal at 37 °C), pH tolerance from 7.0 to 11.0 (optimal at 7.5) and a NaCl concentration ranging from 1.0 to 15.0% (*w*/*v*, optimal at 5.0%). Ubiquinone-8 (Q-8) was the predominant isoprenoid quinone in strain GXY010^T^. The dominant fatty acids (>10%) of strain GXY010^T^ were iso-C_15:0_ (14.65%), summed feature 9 (iso-C_17:1_ *ω*9*c* and/or 10-methyl C_16:0_) (12.41%), iso-C_17:0_ (10.85%) and summed feature 3 (C_16:1_ *ω*7*c* and/or C_16:1_ *ω*6*c*) (10.41%). Phosphatidylethanolamine (PE), phosphatidylglycerol (PG), diphosphatidylglycerol (DPG), unidentifiable glycolipid (GL) and four non-identifiable aminolipids (AL1-AL4) were the predominant polar lipids of strain GXY010^T^. The genomic DNA G+C content was identified as a result of 48.0% for strain GXY010^T^. The strain GXY010^T^ genome consisted of 2,766,857 bp, with 2664 Open Reading Frames (ORFs), including 2586 Coding sequences (CDSs) and 78 RNAs. Strain GXY010^T^ showed Average Nucleotide Identity (ANI) values of 73.4% and 70.6% and DNA–DNA hybridization (DDH) values of 19.2% and 14.5% with reference species *Pseudidiomarina tainanensis* MCCC 1A02633^T^ (=PIN1^T^) and *Pseudidiomarina taiwanensis* MCCC 1A00163^T^ (=PIT1^T^). From the results of the polyphasic analysis, a newly named species, *Pseudidiomarina fusca* sp. nov. within the genus *Pseudidiomarina*, was proposed. The type strain of *Pseudidiomarina fusca* is GXY010^T^ (=JCM 35760^T^ = MCCC M28199^T^ = KCTC 92693^T^).

## 1. Introduction

The genus *Pseudidiomarina* was classified in the family *Idiomarinaceae*, belonging to the order *Alteromonadales* and the class *Gammaproteobacteria.* The description of the family was revised in 2006 by Jean et al. with the introduction of a newly introduced genus, *Pseudidiomarina* [1]. This classification persisted until 2009 when the genus was subsequently merged into the genus *Idiomarina* [2]. The genus *Pseudidiomarina* was subsequently recovered by Liu et al. based on a phylogenetic analysis [3]. The genus *Pseudidiomarina* has been found in diverse environments, including coastal and marine waters, coastal sands, coastal sediments and solar salt flats, all of which are saline areas with an extensive range of salinity [4,5,6,7]. The genus *Pseudidiomarina* currently includes 18 validly published species (https://lpsn.dsmz.de/genus/pseudidiomarina (accessed on 1 December 2023)) [8]. Members of this genus are characterized as chemo-heterotrophic, mesophilic, non-sporulating, Gram-negative rods and iso-branched fatty acids of both 15- and 17-carbon atoms are the predominant components of this genus. Predominant fatty acids are iso-branched and polar lipids include phosphatidylethanolamine (PE), diphosphatidylglycerol (DPG) and phosphatidylglycerol (PG) [9]. In the present study, we describe a bacterium isolated from a sample of surface seawater from the western Pacific Ocean and characterized using polyphasic taxonomic identification. The results indicate that the isolated bacterium is a novel species of the genus *Pseudidiomarina*.

## 2. Materials and Methods

### 2.1. Isolation and Cultivation Conditions

In September 2020, a bacterial strain designated as GXY010^T^ was obtained from the surface seawater of the western Pacific (162°14′ E, 23°13′ N), where the temperature was 28.2 °C and the salinity was 3.45%. The bacterial isolation process involved adding 1 mL of seawater sample to 30 mL of 2216E liquid medium (Qingdao Hope Bio-Technology Co., Ltd., Qingdao, China). The mixture was then incubated at 28 °C for one week for enrichment. Subsequently, three concentration gradients of 10^−2^, 10^−4^ and 10^−6^ were prepared by dilution, and 200 μL of each gradient was spread onto 2216E agar plates (Qingdao Hope Bio-Technology Co., Ltd., Qingdao, China). After incubating the plates at 28 °C until a large number of colonies grew, the purification process was iteratively conducted to isolate a monoclonal strain. Single colonies were picked individually using an inoculation loop and inoculated on pre-prepared test tube slant 2216E agar; all strains, including GXY010^T^, were cultured until moss grew. Preservation solution was prepared with 85% saline (0.85%, *w*/*v*) and 15% glycerol, sterilized and added to the slant medium to rinse the moss and shake it to form a bacterial suspension, which was dispensed into freezing tubes and stored in an ultra-low temperature freezer at −80 °C.

### 2.2. Molecular Analysis

Moore’s method was employed for the extraction of genomic DNA from strain GXY010^T^ [10]. The 16S rRNA gene of strain GXY010^T^ was amplified using a PCR instrument (2720 Thermal Cycler, Applied Biosystems, Foster, CA, USA) and generic bacterial primers (B8F, as well as B1510R) [11]. Pairwise similarity values comparing strain GXY010^T^ with its related reference strains were calculated using the EzBioCloud server (http://www.ezbiocloud.net (accessed on 25 August 2023)), following the method of Yoon et al. [12]. *Pseudidiomarina tainanensis* MCCC 1A02633^T^ and *Pseudidiomarina taiwanensis* MCCC 1A00163^T^ were selected as reference strains and were obtained from the Marine Culture Collection of China (MCCC). We used the EzBioCloud database to obtain the 16S rRNA gene sequences of the relevant strains, and they were analyzed using the Clustal X version 2.0 program [13]. Phylogenetic trees were built using neighbor joining (NJ), maximum likelihood (ML) and maximum parsimony (MP) methods with MEGA X (version 10.2.6) [14,15,16,17]. NJ and ML analyses were conducted using Kimura’s 2-parameter (K2-P) model [18], while MP analysis employed the method TBR (Tree Bisection Reconnection). Phylogenetic tree topologies were assessed using bootstrap resampling and 1000 replicates. *Escherichia coli* ATCC 11775^T^ (GenBank accession: AB681728) was used as an outgroup in all phylogenetic analyses.

### 2.3. Genome Sequencing, Annotation and Analysis

The Illumina HiSeq platform (Illumina, San Diego, CA, USA) was used for whole genome sequencing (WGS). The reads were assembled into sequences using SOAPdenovo2 software (http://sourceforge.net/projects/soapdenovo2/ (accessed on 10 August 2023)) [19]. The genome data of the most similar strain were retrieved from the GenBank database for comparison of genome relatedness. Phylogenetic analyses of the core genomes involved aligning 39 species using MAFFT (version 7.505) software with default settings, followed by trimming to the length of the shortest sequence. Phylogenetic trees of the core genomes were generated using FastTree (version 2.1.10) with JTT+CAT parameters and 1000 bootstrap replicates [20]. *E. coli* ATCC 11775^T^ was used as an outgroup in the phylogenetic analyses. The resulting trees were visualized, annotated and managed using the web-based tool Chiplot (https://www.chiplot.online/ (accessed on 15 October 2023)). Genomic similarity was evaluated using the Average Nucleotide Identity (ANI) value obtained through orthoANI (https://www.ezbiocloud.net/tools/orthoani (accessed on 10 August 2023)). At the same time, genome-to-genome distance calculator (GGDC) software version 2.1 and Formula 2 were employed to calculate DNA–DNA hybridization (DDH) value. Annotation was performed using BLAST+ 2.2.24 and Rapid Annotation using Subsystem Technology (RAST) [21,22]. With reference to Tatusov et al., the Cluster of Orthologous Groups (COG) and Kyoto Encyclopedia of Genes and Genomes (KEGG) databases were utilized for functional gene annotation of GXY010^T^ [23].

### 2.4. Phenotypic Characterization

The shape, size, gloss, margins and color of the colonies on the plates were directly observed and recorded. Referring to the standard method of Beveridge et al. [24], Gram and flagellum staining of strain GXY010^T^ was performed; the results were observed and recorded using a Nikon Eclipse E600 light microscope (Trusco Nakayama Corporation, Shinagawa, Japan). Following staining of strain GXY010^T^ with 1% (*w*/*v*) phosphotungstic acid, cell morphology was observed using transmission electron microscopy (JEM-1200EX, JEOL Ltd., Akishima, Japan) (Figure S1). The experiments to determine the optimum temperature and pH for GXY010^T^ were conducted using 2216E liquid medium, while the assay for optimal salinity was performed using a modified 2216E liquid medium [25], in which K^+^ was substituted for Na^+^ (formulated as 4.78 g K_2_SO_4_, 0.664 g KCl, 0.096 g KBr, 0.04 g SrCl_2_·6H_2_O, 0.0053 g KF, 4.981 g MgCl_2_·6H_2_O, 0.95 g CaCl_2_, 0.228 g KHCO_3_, 0.026 g H_3_BO_3_, 1 L distilled water). The chemical reagents used in the modified 2216E liquid medium were all sourced from Shanghai Sinopharm Chemical Reagent Ltd. in China Shanghai.

The absorbance of the bacterial suspension was measured at 590 nm (OD_590_) to assess the growth of the strain. Three parallel samples were established for each condition of temperature, salinity and pH, and OD_590_ was measured following the experimental procedure. The average value was calculated to identify the optimal conditions for strain growth. In temperature experiments (different temperature, 170 rpm, salinity 3.5%, pH 7.5), cultures were incubated at 0 and 4 °C for 4 weeks with measurements taken every 4 days and at 10, 16, 20, 24, 28, 32, 37, 42 and 45 °C for 1 week and measured daily. For the salinity assay (28 °C, 170 rpm, pH 7.5), the NaCl concentration was varied to 0, 0.5, 1, 2, 2.5, 3, 3.5 and 4 to 15% (*w*/*v*, with 1.0% intervals), and measurements were taken continuously for one week. In the pH range experiment (28 °C, 170 rpm, salinity 3.5%), growth was assessed using a pH buffer system with concentrations set at 20 mM for the following buffers: H_3_PO_4_/KH_2_PO_4_ (pH 2.0), sodium ethanoate/ethanoic acid (pH 3.0–5.0), MES (pH 5.5, 6.0 and 6.5), MOPS (pH 7.0 and 7.5), TRICINE (pH 8.0 and 8.5), TAPS (pH 9.0) and CAPSO (pH 9.5 and 10.0) buffers [26,27]. Measurements were taken continuously for a week.

### 2.5. Enzyme Activity Characterization

Agar media were prepared according to established procedures [28], encompassing media with starch, chitin, casein, gelatin, cellulose, sodium alginate and Tween 20, 40, 80. The only deviation from standard methods was the substitution of distilled water with filtered seawater. To assess the DNase activity of the strains, DNase agar plates from Qingdao Hope Biotechnology were utilized. The degradation efficacy was assessed based on the formation of transparent zones surrounding the colony or when the plates were immersed in the relevant solution. Bacterial cultures were spotted onto enzyme activity agar plates and incubated under optimal conditions. The ability of the strains to hydrolyze Tween 20, 40, 80, chitin and gelatin was assessed by direct observation of the transparent circle surrounding the colonies. Additional enzyme activities were examined using specific identification solutions: Lugol’s iodine solution for amylase activity, 1 M HCl for DNase activity, 3% trichloroacetic acid for caseinase activity, 1 mg/mL Congo red solution with 1 M NaCl for cellulase activity and 10% CaCl_2_ for sodium alginate enzyme activity. Oxidase activity was tested using 1% tetramethyl-p-phenylenediamine dihydrochloride (TMPD), and catalase activity was assessed using 3% hydrogen peroxide solution. Using API 20E, 20NE and ZYM test strips (bioMérieux Shanghai Ltd., Shanghai, China), as well as GEN III MicroPlates (Biolog Inc., Hayward, CA, USA), the constitutive enzyme activities, fermentation/oxidation profile, acid production and the utilization rate of substrates as the sole carbon and energy source were determined for GXY010^T^ following the manufacturer’s instructions.

### 2.6. Chemotaxonomy

During the exponential phase, GXY010^T^ and its reference strains were cultivated concurrently in 2216E liquid medium and subsequently harvested for the analysis of cellular fatty acid types and contents using the method reported by Komagata and Suzuki [29]. Fatty acid methyl esters were extracted and analyzed following the standard MIDI protocol, and the TSBA 6.0 database was employed for identification [30]. After incubation in 2216E liquid medium, GXY010^T^ and the reference strains were harvested, lyophilized and subjected to analysis for respiratory quinones and polar lipids. Respiratory quinones were extracted from the strains using chloroform/methanol (2:1, *v*/*v*), separated via TLC and identified through LC-MS [31]. The polar lipids were separated using the two-dimensional TLC method on 60 F254 silica gel plates (Merck KGaA, Darmstadt, Germany), following Minnikin et al.’s method [32]. The first-dimensional solution consisted of chloroform/methanol/water in a volume ratio of 65:25:4, while the second-dimensional solution comprised chloroform/methanol/acetic acid/water at a volume ratio of 80:12:15:4 [33]. Additionally, the identified extracted lipids were characterized by spraying the plates with suitable detection reagents for amino groups (ninhydrin/n-butyl alcohol, Dragendoff’s reagent), glycolipids (alpha-naphthol/sulfuric acid) and total lipids (molybdatophosphoric acid).

### 2.7. Repositories

The GenBank 16S rRNA gene and genome sequence accession numbers for strain GXY010^T^ are OP351365 and JANFPJ000000000, respectively.

## 3. Results

### 3.1. Physiological Characteristics

GXY010^T^ thrived under aerobic conditions on 2216E agar plates, displaying colonies that were smooth, rounded and light brown in color. The cells were Gram-staining negative and non-motile with a lack of flagella. The strains were grown at temperatures ranging from 10 to 42 °C (optimum 37 °C). After 72 h of incubation on 2216E agar at 37 °C, the cell morphology was observed to be rod-shaped, with a length of about 2.0 to 3.0 μm and a width of about 0.4 to 0.6 μm. Strain GXY010^T^ was able to grow at a salinity of 1.0% to 15.0% (*w*/*v*) NaCl (optimal salinity of 5.0%) and a pH of 7.0 to 11.0 (optimal pH of 7.5). The morphological, physiological and biochemical characteristics of GXY010^T^ are summarized in Table 1. The strain demonstrated oxidase and catalase activities and showed the ability to hydrolyze Tween 20, Tween 40, gelatin and DNA. The following constitutive enzyme activities were positive in API ZYM tests: esterase (C4), esterase lipase (C8), lipase (C14), valine arylamidase, cystine arylamidase, trypsin, α-chymotrypsin, acid phosphatase, α-galactosidase, β-galactosidase, β-glucuronidase, α-glucosidase, β-glucosidase, N-acetyl-β-glucosaminidase, α-mannosidase and β-fucosidase. The results of the enzyme activity assay demonstrated that strains GXY010^T^, *P. tainanensis* MCCC 1A02633^T^, *P. taiwanensis* MCCC 1A00163^T^ and *Pseudidiomarina marina* PIM1^T^ were positive for oxidase, catalase, esterase (C4), esterase lipase (C8), lipase (C14), valine arylamidase, cystine arylamidase, trypsin, α-chymotrypsin and acid phosphatase. However, these strains tested negative for the hydrolysis of casein, chitin, starch, cellulose and sodium alginate.

The results of API 20E and 20NE strips were positive for a reduction in acetoin (Voges–Proskauer reaction) and activity of urease and arginine dihydrolase; they were negative for a reduction in nitrate, utilization of citric acid, production of H_2_S, indole, the activity of β-galactosidase, gelatinase, lysine decarboxylase, ornithine decarboxylase, tryptophan deaminase and β-glucosidase, assimilation of d-glucose, l-arabinose, mannose, mannitol, N-acetyl-glucosamine, maltose, potassium gluconate, capric acid, adipic acid, malic acid, trisodium citrate and phenylacetic acid, and the fermentation of glucose, mannitol, inositol, sorbitol, rhamnose, sucrose, melibiose, amygdalin and arabinose.

The results of Biolog GEN III microplates were positive for utilization of dextrin, d-glucose-6-PO_4_, d-fructose-6-PO_4_, p-hydroxy-phenylacetic acid, l-lactic acid, d-glucuronic acid, Tween 40, acetoacetic acid, d-malic acid, l-malic acid, propionic acid and acetic acid; they were negative for utilization of d-maltose, gentiobiose, d-turanose, d-fructose, d-galactose, mucic acid, 3-methyl glucose, d-fucose, l-fucose, glycyl-l-proline, l-arginine, α-keto-butyric acid, l-aspartic acid, l-glutamic acid, d-galacturonic acid, l-galactonic acid lactone, l-rhamnose, d-trehalose, d-melibiose, d-cellobiose, sucrose, stachyose, d-raffinose, α-d-lactose, β-methyl-d-glucoside, d-salicin, N-acetyl-d-glucosamine, N-acetyl-β-d-mannosamine, α-d-glucose, N-acetyl-d-galactosamine, N-acetyl neuraminic acid, d-mannose, inosine, d-sorbitol, d-mannitol, d-arabitol, myo-inositol, glycerol, d-aspartic acid, d-serine, gelatin, d-alanine, l-histidine, l-pyroglutamic acid, l-serine, pectin, d-gluconic acid, glucuronamide, quinic acid, d-saccharic acid, methyl pyruvate, d-lactic acid methyl ester, citric acid, α-keto-glutaric acid, bromo-succinic acid, γ-amino-butryric acid, α-hydroxy-butyric acid, formic acid and β-hydroxy-d,l-butyric acid.

### 3.2. Chemotaxonomic Characteristics

The dominant fatty acids (>10%) of strain GXY010^T^ were iso-C_15:0_ (14.65%), summed feature 9 (iso-C_17:1_ *ω*9*c* and/or 10-methyl C_16:0_) (12.41%), iso-C_17:0_ (10.85%) and summed feature 3 (C_16:1_ *ω*7*c* and/or C_16:1_ *ω*6*c*) (10.41%). Moreover, compared to the two reference strains, strain GXY010^T^ had higher amounts of C_17:0_, anteiso-C_17:0_, C_17:1_ *ω*8*c* and summed feature 3 (C_16:1_*ω*7*c* or/and C_16:1_*ω*6*c*) but less iso-C_15:0_ and iso-C_15:1_ F. Table 2 provides a detailed comparison of the cellular fatty acid type and content between strain GXY010^T^ and the reference strains. Ubiquinone-8 (Q-8) was determined to be the abundant isoprenoid quinone in strain GXY010^T^, the same as other strains in the family *Idiomarinaceae*. Figure S5a illustrates that the primary polar lipids of strain GXY010^T^ include phosphatidylethanolamine (PE), phosphatidylglycerol (PG), diphosphatidylglycerol (DPG), unidentified glycolipid (GL) and four non-identified aminolipids (AL1-AL4). In comparison, the major polar lipids of strain *P. tainanensis* MCCC 1A02633^T^ were PE, PG, DPG and four non-identified aminolipids (AL1-AL4), as depicted in Figure S5b. Figure S5c displays that the primary polar lipids of strain *P. taiwanensis* MCCC 1A00163^T^ include PE, PG, DPG and three non-identified aminolipids (AL1-AL3). A distinctive feature observed in the polar lipids of strain GXY010^T^ is the presence of an unidentified glycolipid (GL), setting it apart from the other two reference strains.

### 3.3. 16S rRNA Gene Sequences Analysis

**Figure 1:**
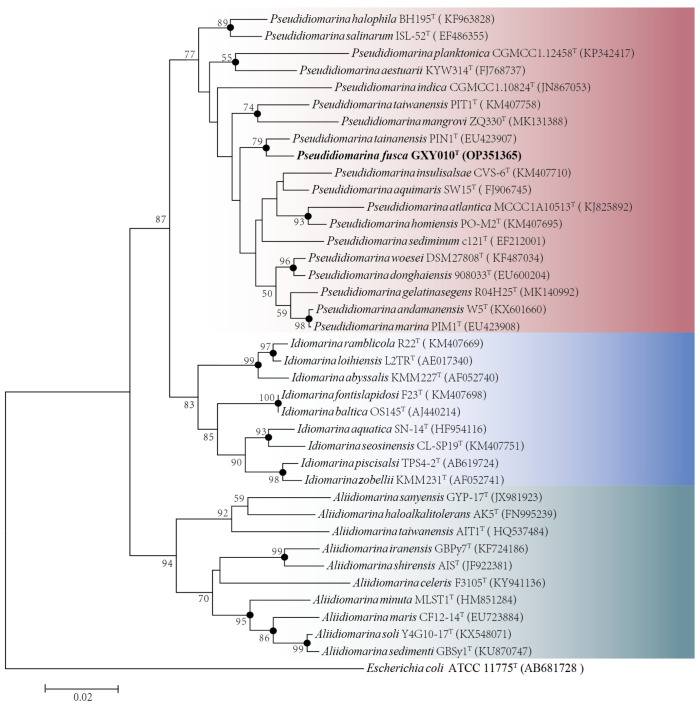
Neighbor-joining phylogenetic tree based on 16S rRNA gene sequences (1505 bp) showing the phylogenetic position of strain GXY010^T^ and other closely related species. Percentage bootstrap values above 50% (1000 replicates) are shown at branch nodes. Closed circles indicate that the corresponding nodes were also recovered in trees generated with the neighbor-joining, maximum-likelihood and maximum-parsimony algorithms. Red, *Pseudidiomarina* genus. Blue, *Idiomarina* genus. Green, *Aliidiomarina* genus. *Escherichia coli* ATCC 11775^T^ (GenBank accession: AB681728) was used as the outgroup. The putative novel genospecies are highlighted in bold. Bar, 0.02 nucleotide substitutions per nucleotide position.

Based on the complete sequence of the 16S rRNA gene (1505 bp) of the strain GXY010^T^, pairwise alignment exhibited the largest sequence similarity of 98.15% and 97.81% to *P. tainanensis* MCCC 1A02633^T^ and *P. marina* PIM1^T^, respectively. Phylogenetic analysis on the basis of the NJ (Figure 1), ML (Figure S2) and MP (Figure S3) algorithms indicated that strain GXY010^T^ formed a close branch with *P. tainanensis* MCCC 1A02633^T^. From the 16S rRNA gene identification and phylogenetic analysis, parallel experiments were conducted using *P. tainanensis* MCCC 1A02633^T^ and *P. taiwanensis* MCCC 1A00163^T^ from the MCCC as reference strains. In the 16S rRNA gene-based tree of the family *Idiomarinaceae*, the genera *Pseudidiomarina* and *Idiomarina* formed sister groups, with the genus *Aliidiomarina* being more closely related to them.

### 3.4. Genome Analysis

**Figure 2:**
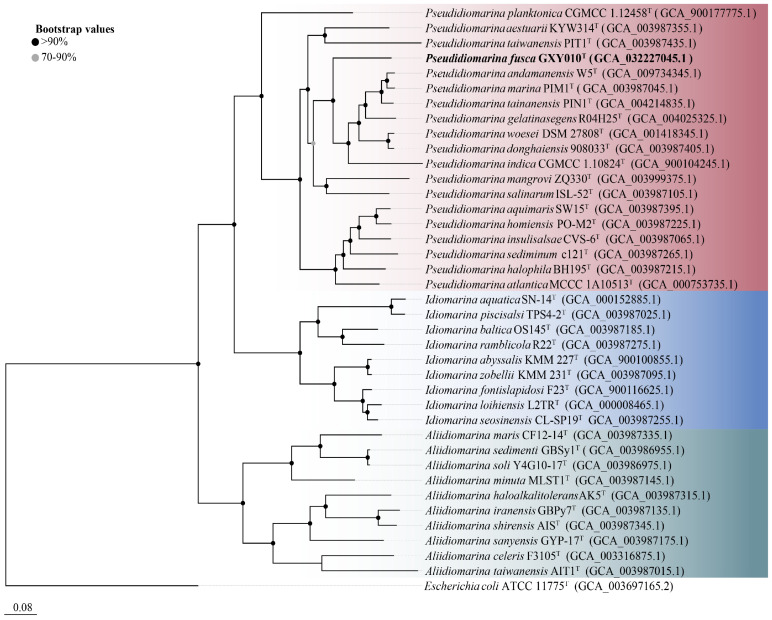
Core genome-based phylogenetic tree of the family *Idiomarinaceae* inferred using FastTree 2.1.10 with JTT+CAT parameters and 1000 bootstrap replicates and rooted using *Escherichia coli* ATCC 11775^T^. Bootstrap values are indicated at the nodes of the solid circle filled with different colors. The putative novel genospecies are highlighted in bold. Red, *Pseudidiomarina* genus. Blue, *Idiomarina* genus. Green, *Aliidiomarina* genus. Genome accession numbers are indicated in parentheses. Bar, 0.08 substitutions per position.

The genome of strain GXY010^T^ is composed of 2,766,857 bp, housing 2664 ORFs, which encompass 2586 CDSs and 78 RNAs (71 tRNAs, 3 rRNAs and 4 ncRNAs). Detailed draft genomic characteristics of strain GXY010^T^ and reference strains can be found in Table S1. The core genome-based phylogenetic tree aligns with the relationships observed in the 16S rRNA gene-based tree, placing GXY010^T^ within the genus *Pseudidiomarina* (Figure 2). WGS results indicate that the genomic DNA of strain GXY010^T^ has a G+C content of 48.0% and is classified within the genus *Pseudidiomarina* (47.2–53.0%). The ANI values between strain GXY010^T^ and reference species *P. tainanensis* MCCC 1A02633^T^ and *P. taiwanensis* MCCC 1A00163^T^ were 73.4% and 70.6%, respectively, which were designated below the threshold of 95–96% [34]. Similarly, the digital DDH values between strain GXY010^T^ and reference species *P. tainanensis* MCCC 1A02633^T^ and *P. taiwanensis* MCCC 1A00163^T^ were 19.2% and 14.5%, respectively, which were well below the recognized threshold of 70% for establishing a novel species [35].

Strain GXY010^T^, along with *P. tainanensis* MCCC 1A02633^T^ and *P. taiwanensis* MCCC 1A00163^T^, underwent annotation using the COG database. As shown in Figure S4, strain GXY010^T^ had a higher relative abundance of K (Transcription), L (Replication, recombination and repair), V (Defense mechanisms), T (Signal transduction mechanisms) and S (Function unknown), and lower relative abundance of A (RNA processing and modification), J (Translation, ribosomal structure and biogenesis), D (Cell cycle control, cell division, chromosome partitioning), O (Posttranslational modification, protein turnover, chaperones), C (Energy production and conversion), E (Amino acid transport and metabolism), F (Nucleotide transport and metabolism), H (Coenzyme transport and metabolism) and I (Lipid transport and metabolism) than its reference strains. Based on the KEGG database, glutamate synthase (NADPH/NADH) (EC 1.4.1.13) and alcohol dehydrogenase (EC 1.1.1.1) were found in strain GXY010^T^, whilst it was not found in *P. tainanensis* MCCC 1A02633^T^ and *P. taiwanensis* MCCC 1A00163^T^. The genome of strain GXY010^T^ features a gene encoding octaprenyl diphosphate synthase (EC 2.5.1.90), indicating its capacity for synthesizing ubiquinone-8 (Q-8), the most abundant respiratory quinone in the family *Idiomarinaceae*. Additionally, it harbors genes responsible for cardiolipin synthase (EC 2.7.8.41), facilitating DPG production and phosphatidylserine decarboxylase (EC 4.1.1.65), enabling PE synthesis. Along with PG, these constitute the major identified polar lipids of the genus *Pseudidiomarina*.

## 4. Conclusions

The enzyme activities of strain GXY010^T^ share similarities with other reference bacteria, as all strains exhibit positive results for oxidase and catalase tests. However, a notable point of distinction is that only GXY010^T^ demonstrates the ability to hydrolyze gelatin. While all strains share the same major fatty acid types, there are variations in their respective content percentages. Strain GXY010^T^ possesses a unique, unidentifiable glycolipid (GL) absent in other strains, further distinguishing it from other members of the genus *Pseudidiomarina* in terms of polar lipid composition. In summary, the GXY010^T^ strain can be distinguished from the reference strains by several phenotypic features, including both morphological and chemical taxonomic markers. The 16S rRNA phylogenetic tree, core genome-based phylogenetic tree and whole-genome indices (ANI and DDH) collectively validate that strain GXY010^T^ is affiliated with the genus *Pseudidiomarina* and exhibits noticeable distinctions from other species within the genus. The alignment of evidence from phenotypic, phylogenetic and genetic data unequivocally designates strain GXY010^T^ as a novel species within the genus *Pseudidiomarina*, leading to the formal proposal of the name *Pseudidiomarina fusca* sp. nov. A description of *Pseudidiomarina fusca* sp. nov. can be found below.

*Pseudidiomarina fusca* (fus’ca. L. fem. adj. *fusca*, brown): The strain grew aerobically on 2216E agar plates, the colonies were light brown, smooth and rounded, the cell was non-motile and without flagellum, and the cell Gram staining result was negative. Strain GXY010^T^ grew most optimally at 37 °C, pH 7.5 and salinity 5.0%. It showed positive activities for oxidase and catalase. Hydrolysis activities were observed for Tween 20, Tween 40, gelatin and DNA, but not for Tween 80, chitin, sodium alginate, cellulose, casein and starch. The principal respiratory quinone was ubiquinone-8 (Q-8). The dominant fatty acids (>10%) of strain GXY010^T^ were iso-C_15:0_, summed feature 9 (iso-C_17:1_ *ω*9*c* and/or 10-methyl C_16:0_), iso-C_17:0_ and summed feature 3 (C_16:1_ *ω*7*c*/C_16:1_ *ω*6*c*). The polar lipids consisted mainly of PE, PG, DPG, unidentified GL and four unidentified ALs. The species is affiliated with the genus *Pseudidiomarina* of the family *Idiomarinaceae*. The DNA G+C content of the type strain is 48.0%.

The type strain, GXY010^T^ (=JCM 35760^T^ = MCCC M28199^T^ = KCTC 92693^T^), was isolated from the surface seawater of the western Pacific Ocean. The GenBank accession number for the 16S rRNA gene sequence of GXY010^T^ is OP351365. The WGS project of strain GXY010^T^ has been deposited in GenBank under the accession number JANFPJ000000000. The version presented in this paper is the JANFPJ000000000 version.

## Figures and Tables

**Table 1:** Different characteristics between strain GXY010^T^ and the reference strains.

Characteristics	1	2	3	4
Colony color	light brown	yellow	non-pigment	gray-white
Cell size (μm)	2.0−3.0 × 0.4−0.6	2.5−3.2 × 0.6−0.9	0.8−2.4 × 0.6−0.8	2.5−3.0 × 0.5−0.9
Temperature (°C)	10–42	10–42	15–42	10–42
(optium Temperature)	(37)	(35)	(35)	(30–35)
NaCl (%)	1.0–15.0	0–15.0	0.5–11.0	0.5–15.0
(optimum NaCl%)	(5.0)	(5.0)	(2.5)	(2.0–5.0)
pH	7.0–11.0	6.0–10.0	6.5–10.0	6.0–10.0
(optimum pH)	(7.5)	(7.5)	(8.0)	(7.0–8.0)
Enzyme activity				
Oxidase	+	+	+	+
Catalase	+	+	+	+
Tween 20	+	+	+	−
Tween 40	+	+	−	−
Tween 80	−	+	−	−
DNase	+	−	−	+
Gelatin	+	−	−	−
DNA G+C content (%) *	48.0	46.9	49.3	46.6

Strains: (1) GXY010^T^; (2) *Pseudidiomarina tainanensis* MCCC 1A02633^T^; (3) *Pseudidiomarina taiwanensis* MCCC 1A00163^T^; (4) *Pseudidiomarina marina* PIM1^T^ (Data from Ref. [4]). Data were obtained in this study unless indicated otherwise. +, positive; −, negative. * Data from draft genomes, collected from the NCBI Genome database.

**Table 2:** Cellular fatty acid content (%) of strain GXY010^T^ and reference strains.

Fatty Acid	1	2	3
C_14:0_	1.16	TR	3.46
C_16:0_	7.01	4.00	10.62
C_17:0_	1.48	TR	1.05
C_17:0_ cyclo	−	1.65	−
C_18:0_	3.63	3.06	5.24
iso-C_15:0_	**14.65**	18.98	15.50
iso-C_17:0_	**10.85**	13.42	5.31
iso-C_15:1_ F	2.21	3.09	4.82
anteiso-C_15:0_	1.31	TR	-
anteiso-C_16:0_	1.23	TR	1.37
anteiso-C_17:0_	1.51	1.14	1.47
C_17:1_ *ω*8*c*	1.76	1.12	TR
C_12:0_ 3-OH	1.16	TR	2.01
iso-C_11:0_ 3-OH	4.54	6.77	5.13
iso-C_13:0_ 3-OH	3.37	3.48	2.43
Summed features 3	**10.41**	5.62	10.29
Summed features 5	1.25	TR	1.29
Summed features 9	**12.41**	12.09	5.20

Strains: (1) GXY010^T^; (2) *P. tainanensis* MCCC 1A02633^T^; (3) *P. taiwanensis* MCCC 1A00163^T^. Data were obtained in this study unless indicated otherwise. Only fatty acids > 1% among the total fatty acids of at least one of the strains are shown. Bold type indicates major fatty acids (>10%). TR, trace amount (<1% of total). −, not detected. Summed Features are fatty acids that cannot be resolved reliably from another fatty acid using the chromatographic conditions chosen. The MIDI system groups these fatty acids together as one feature with a single percentage of the total. Summed feature 3 comprises C_16:1_ *ω*6c and/or C_16:1_ *ω*7*c*. Summed feature 5 comprises anteiso-C_18:0_ and/or C_18:2_ *ω*6,9*c*. Summed feature 9 comprises iso-C_17:1_ *ω*9*c* and/or 10-methyl C_16:0_.

## Data Availability

All data generated or analyzed during this study are included.

## Supplementary Materials

The following supporting information can be downloaded at: https://www.mdpi.com/article/10.3390/microorganisms12020408/s1, Table S1: Genome features of strain GXY010^T^ and strains of the genus *Pseudidiomarina*; Figure S1: Transmission electron micrograph of a negatively stained cell of GXY010^T^; Figure S2: Maximum-likelihood phylogenetic tree based on 16S rRNA gene sequences (1505 bp) showing the phylogenetic position of strain GXY010^T^ and other closely related species; Figure S3: Maximum-parsimony phylogenetic tree based on 16S rRNA gene sequences (1505 bp) showing the phylogenetic positions of strain GXY010^T^ and other closely related species; Figure S4: Comparison of gene content between GXY010^T^ and their reference strains; Figure S5: Total polar lipids of strain GXY010^T^ and the reference strain were separated by two-dimensional TLC and detected with 10% ethanolic molybdophosphoric acid.
