# Supplementary material for: Pseudidiomarina fusca sp. nov., Isolated from the Surface Seawater of the Western Pacific Ocean
Source: Microorganisms. 2024 Feb 18;12(2):408. doi: 10.3390/microorganisms12020408 (PMC10891523; doi:10.3390/microorganisms12020408)

Supplementary Materials

Pseudidiomarina fusca sp. nov., Isolated from Surface Seawater of the Western Pacific Ocean

Yaru Wang[1, 2, 3], Xiaolei Wang[1, 2, 3], Xueyu Gao[1, 2, 3], Jingjing He[1, 2, 3], Xiaoyu Yang[1, 2, 3], Yunxiao Zhang[1, 2, 3], Xiao-Hua Zhang[1, 2, 3] and Xiaochong Shi [1, 2, 3*]

[1] Frontiers Science Center for Deep Ocean Multispheres and Earth System, and College of Marine Life Sciences, Ocean University of China, Qingdao 266003, P. R. China

[2] Laboratory for Marine Ecology and Environmental Science, Laoshan Laboratory, Qingdao 266237, P. R. China

[3] Institute of Evolution & Marine Biodiversity, Ocean University of China, Qingdao 266003, P. R. China

***Author for Correspondence:**

Xiaochong Shi, College of Marine Life Sciences, Ocean University of China, 5 Yushan Road, Qingdao 266003, P. R China, Tel/Fax: +86-532-82032721, Email: shixiaochong@ouc.edu.cn

Subject category: New taxa–*Proteobacteria*

Running title: *Pseudidiomarina fusca* sp. nov.

Table S1. Genome features of strain GXY010^T and strains of the genus *Pseudidiomarina*.

Strains	Genome size (Mb)	G+C content (%)	rRNA	tRNA	Contig No.	Contig N50 (bp)	Genbank accession No.
Pseudidiomarina fusca GXY010^T	2.80	48.0	3	71	117	129,818	PRJNA860053
P. tainanensis PIN1^T	2.37	47.4	7	49	5	1,762,872	PRJNA420138
P. taiwanensis PIT1^T	2.20	49.3	11	49	7	591,787	PRJNA420139
P. marina PIM1^T	2.41	47.2	7	52	10	595,684	PRJNA420132
P. sediminum c121^T	2.67	50.3	6	53	7	1,473,225	PRJNA420136
P. homiensis PO-M2^T	2.61	50.0	6	50	6	1,900,389	PRJNA420126
P. mangrovi ZQ330^T	2.50	51.0	3	45	22	343,341	PRJNA504364
P. gelatinasegens R04H25^T	2.47	48.2	4	52	30	378,411	PRJNA504716
P. aquimaris SW15^T	2.80	50.2	11	50	22	254,670	PRJNA419972
P. halophila BH195^T	2.62	50.6	4	49	10	656,066	PRJNA420125
P. insulisalsae CVS-6^T	2.56	52.3	8	51	19	207,837	PRJNA420127
P. aestuarii KYW314^T	2.64	49.1	3	49	12	1,928,469	PRJNA419973
P. planktonica CGMCC 1.12458^T	2.57	49.0	3	51	6	1,833,841	PRJNA370089
P. atlantica MCCC 1A10513^T	2.70	50.2	4	52	49	250,685	PRJNA253896
P. donghaiensis 908033^T	2.57	48.1	3	49	15	334,465	PRJNA419975
P. indica CGMCC 1.10824^T	2.20	49.5	12	47	32	139,063	PRJNA255599
P. salinarum ISL-52^T	2.48	53.0	4	51	5	1,814,714	PRJNA420135
P. andamanensis W-5^T	2.40	47.0	12	56	1	2,414,243	PRJNA490609
P. woesei DSM 27808^T	2.44	47.8	7	48	17	317,631	PRJNA288988

Figure S1 Transmission electron micrograph of a negatively stained cell of GXY010^T. Bar, 500 nm

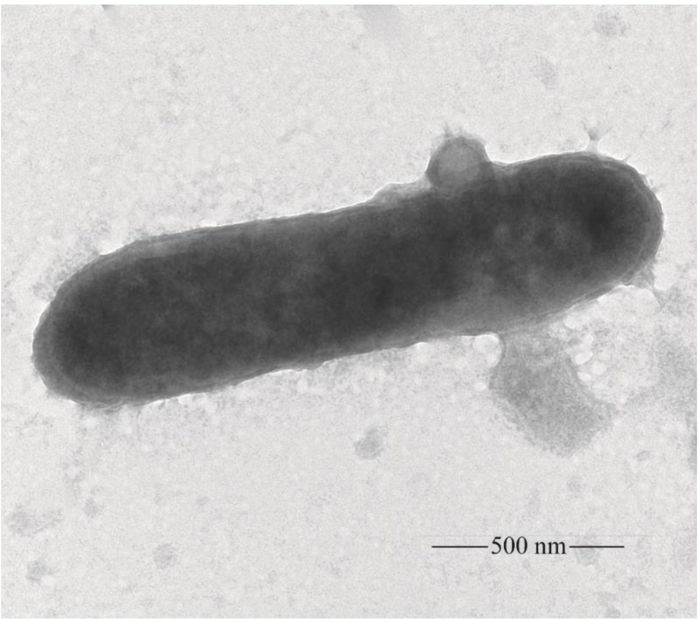

Figure S2 Maximum-likelihood phylogenetic tree based on 16S rRNA gene sequences (1,505 bp) showing the phylogenetic position of strain GXY010^{T} and other closely related species. Percentage bootstrap values above 50% (1,000 replicates) are shown at branch nodes. *Escherichia coli* ATCC 11775^{T} (GenBank accession: AB681728) was used as the outgroup.

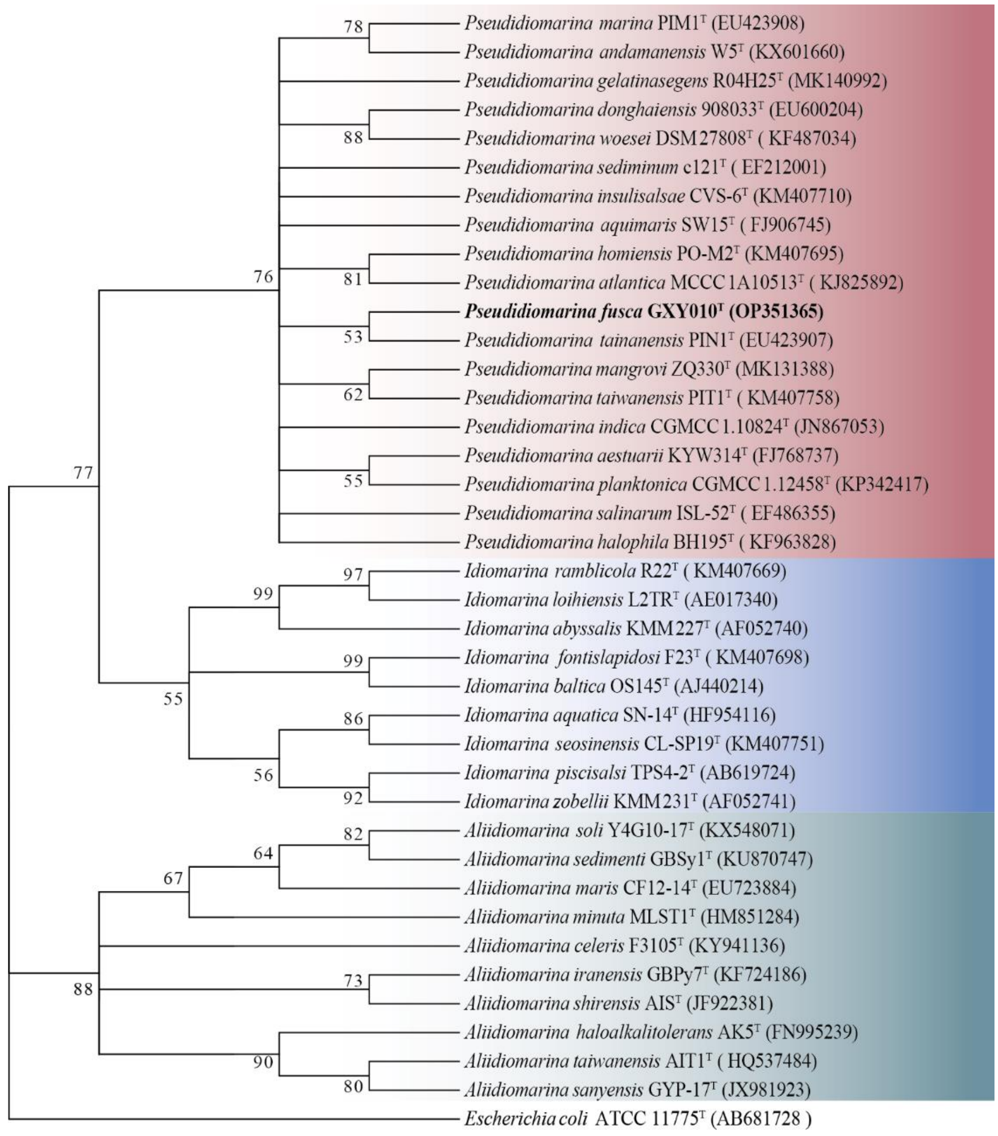

Figure S3 Maximum-parsimony phylogenetic tree based on 16S rRNA gene sequences (1,505 bp) showing the phylogenetic positions of strain GXY010^{T} and other closely related species. Percent bootstrap values above 50% (1,000 replicates) are shown at branch nodes. *Escherichia coli* ATCC 11775^{T} (GenBank accession: AB681728) was used as the outgroup.

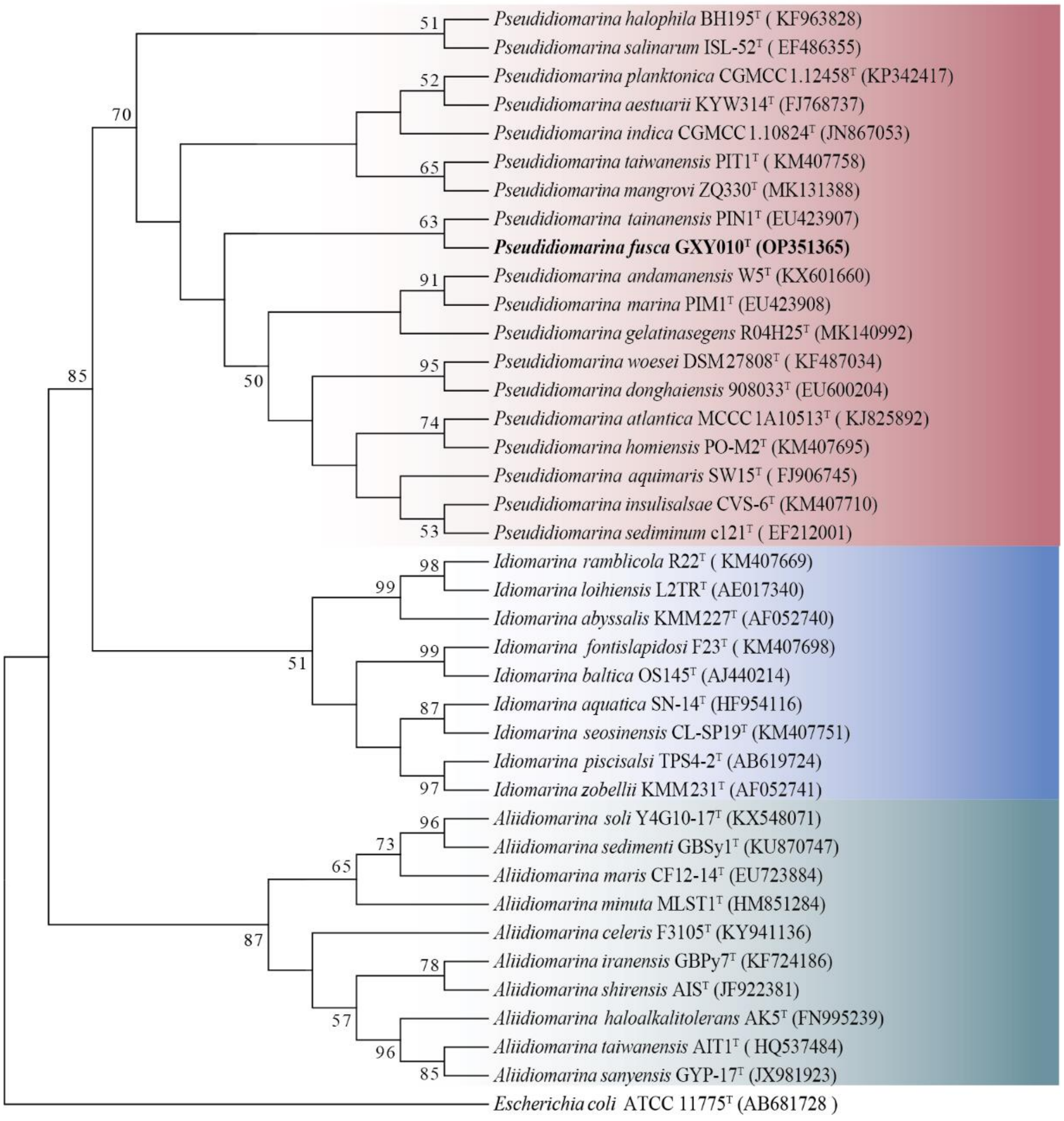

Figure S4. Comparison of gene content between GXY010^T and their reference strains. Relative abundance compared to all COG categories of the orthologous and specific genes in each strain. COG functional categories are described as follows: A, RNA processing and modification; B, Chromatin structure and dynamics; C, Energy production and conversion; D, Cell cycle control, cell division, chromosome partitioning; E, Amino acid transport and metabolism; F, Nucleotide transport and metabolism; G, Carbohydrate transport and metabolism; H, Coenzyme transport and metabolism; I, Lipid transport and metabolism; J, Translation, ribosomal structure and biogenesis; K, Transcription; L, Replication, recombination and repair; M, Cell wall/membrane/envelope biogenesis; N, Cell motility; O, Posttranslational modification, protein turnover, chaperones; P, Inorganic ion transport and metabolism; Q, Secondary metabolites biosynthesis, transport and catabolism; R, General function prediction only; S, Function unknown; T, Signal transduction mechanisms; U, Intracellular trafficking, secretion, and vesicular transport; V, Defense mechanisms; W, Extracellular structures; Z, Cytoskeleton.

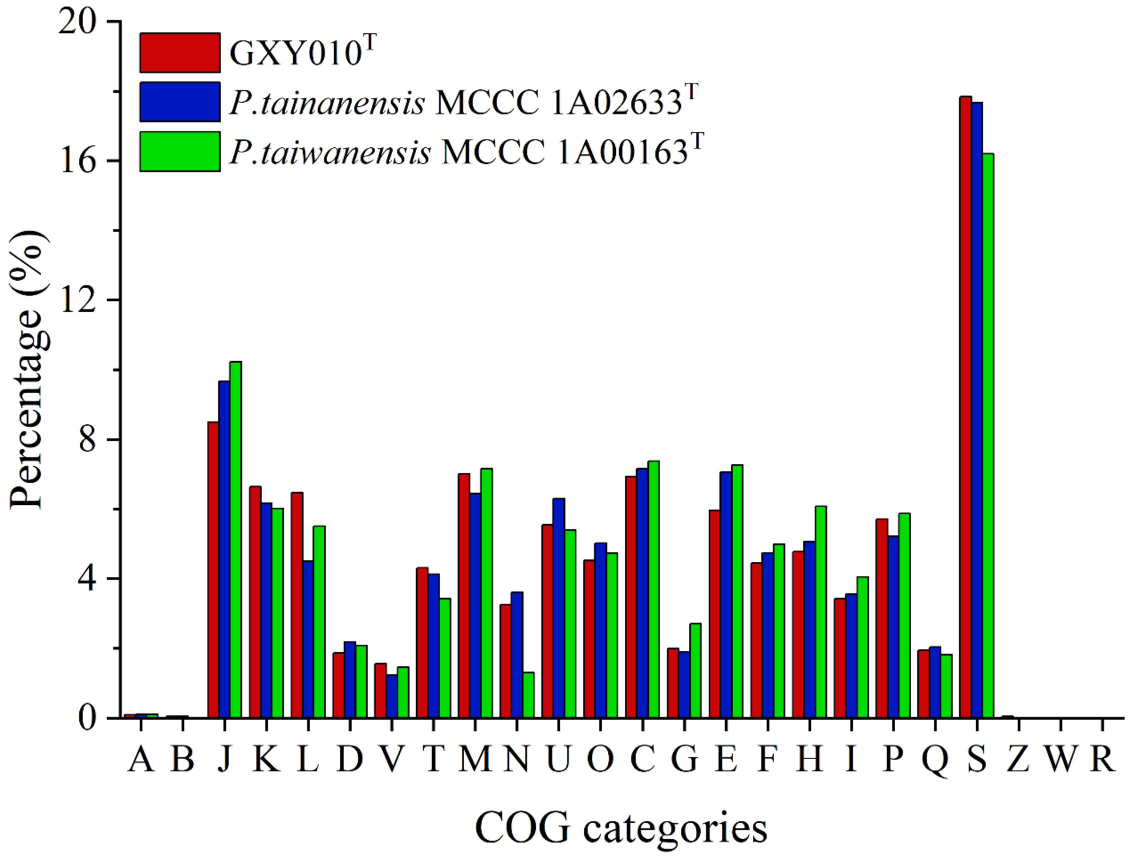

Figure S5 Total polar lipids of strain GXY010^T and the reference strain were separated by two-dimensional TLC and detected with 10% ethanolic molybdophosphoric acid. PE, phosphatidylethanolamine; PG, phosphatidylglycerol; DPG, diphosphatidylglycerol; GL, unidentified glycolipid; AL, unidentified aminolipids.

Strains: a. GXY010^T; b. *P. tainanensis* MCCC 1A02633^T; c. *P. taiwanensis* MCCC 1A00163^T

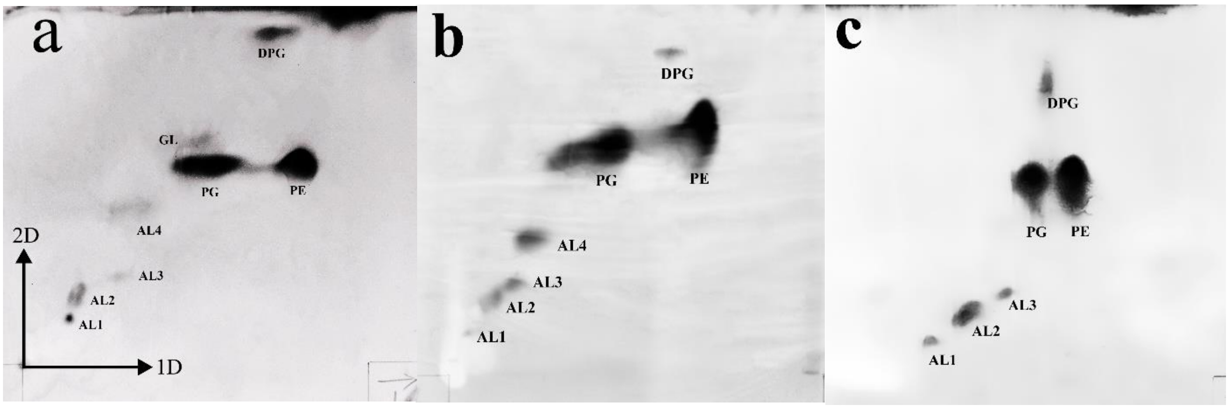

Supplement: Supplementary file 1 [file microorganisms-12-00408-s001.zip › microorganisms-2842162-supplementary.pdf]
